# Testing Causal Effects of Empathy on Children’s Prosociality in Politeness Dilemmas - An Intervention Study

**DOI:** 10.1162/opmi_a_00102

**Published:** 2023-09-20

**Authors:** Noemi Thiede, Roman Stengelin, Astrid Seibold, Daniel B. M. Haun

**Affiliations:** Department of Comparative Cultural Psychology, Max Planck Institute for Evolutionary Anthropology, Leipzig, Germany; Department of Psychology and Social Work, University of Namibia, Windhoek, Namibia; Independent Researcher, Leipzig, Germany

**Keywords:** empathy, polite lies, prosocial lies, empathy task, empathy intervention, prosociality

## Abstract

Empathy is commonly considered a driver of prosociality in child ontogeny, but causal assumptions regarding this effect mostly rely on correlational research designs. Here, 96 urban German children (5–8 years; 48 girls; predominantly White; from mid-to-high socioeconomic backgrounds) participated in an empathy intervention or a control condition before prosocial behaviors (polite lie-telling: rating the drawing as *good*; prosocial encouragement: utterances interpreted as cheering up the artist) were assessed in an art-rating task. Contrasting children’s empathy at baseline with their empathy after the intervention indicated promoted empathy compared to the control group. Despite the intervention’s effect on children’s empathy, there were no simultaneous changes in prosocial behaviors. At the same time, children’s empathy at baseline was associated with their prosocial encouragement. These results indicate conceptual associations between children’s empathy and prosociality. However, they do not support strict causal claims regarding this association in middle childhood. Further applications of the novel short-time intervention to address causal effects of empathy on prosociality and other developmental outcomes are discussed.

## INTRODUCTION

One of the defining characteristics of the human species is our tendency not only to be social but prosocial. Understanding the motivational underpinnings of prosocial behavior as it unfolds in childhood is thus of particular interest for developmental psychology research (Dahl & Brownell, 2019; Hepach & Warneken, 2018). Empathy, the capacity to apprehend and feel the same or similar emotions as others, has frequently been considered essential in this regard (Eisenberg & Miller, 1987; Vaish, 2020) by evoking an altruistic motivation to support and benefit those we empathize with. Following Hoffman (2000), we conceptualize empathy as comprising affective components, such as the emotional experience of others’ feelings (i.e., emotional contagion), and cognitive components, including self-other differentiation or perspective-taking.

Research suggests that children’s empathy and prosociality are intertwined (Eisenberg et al., 2006; Eisenberg & Miller, 1987). From their second year of life, children ”transform the early developing affective experience of empathy to a more sympathetic, other-focused experience” (McDonald & Messinger, 2011, p. 5). From this age, children’s capacities to understand and feel with others may lead them to uplift others’ emotions consequentially (Grossmann, 2018; Hepach et al., 2013; Vaish & Warneken, 2012).

For empathy to foster prosocial behavior, individuals must identify how to benefit their social partners in a given situation. This decision may be straightforward in some cases (i.e., helping someone in distress) but much more challenging in others. For example, some politeness contexts urge individuals to ponder two fundamental communication norms: While the *Maxim of Quality* (Grice, 1995) encourages individuals to communicate truthfully and informatively, the *Politeness Principle* (Leech, 1983) prescribes an amicable attitude not to hurt or harm interlocutors. To follow the latter principle, politeness lies may help protect the other’s feelings and avoid conflict while harming the *Maxim of Quality* (for an overview, see Giles et al., 2019)

Developmental research suggests that children start telling polite lies around age three (Talwar & Lee, 2002; Talwar et al., 2007). In the following years, their polite lie-telling becomes more frequent and sophisticated (Popliger et al., 2011; Talwar & Crossman, 2011; Talwar et al., 2007; Warneken & Orlins, 2015). With age, children increasingly consider the social context when deciding whether to tell polite lies (Fu & Lee, 2007; Warneken & Orlins, 2015). Testing the prosocial character of polite lie-telling in childhood, Warneken and Orlins (2015) varied the mood of an adult artist, who claimed to be either sad or neutral about their drawing. Whereas younger children were indifferent to the artist’s emotional states, children around the age of seven started to lie selectively when facing sad artists (Warneken & Orlins, 2015). Thus, from middle childhood, polite lies are genuinely prosocial acts “to make others feel better” (Warneken & Orlins, 2015, p. 259; see also Jakubowska et al., 2021).

Thus, empathy has been discussed as a potential driver of polite lie-telling in child development (Popliger et al., 2011; Talwar & Crossman, 2011). In a seminal study, Nagar et al. (2020) assessed children’s empathy via parental questionnaires (Dadds et al., 2008) and induced children’s empathy by manipulating the affective states of children’s interaction partners. In a rigged board game, children’s partners reported being sad or neutral due to their weak game performance. At some point, they asked the child to falsely state that they, but not the child, had won the previous round (see also Talwar et al., 2017). Children told more lies when their partner had acted sadly than neutrally hitherto, but this effect was only evident if lying came at no cost to the child (i.e., losing a prize). Interestingly, parent-reported cognitive empathy correlated with children’s prosocial lie-telling.

Demedardi et al. (2021) linked parent-rated empathy of four- to eleven-year-old French children (Dadds et al., 2008) and their prosocial lie-telling in a gaming context similar to that of Nagar et al. (2020). Here, parent-rated empathy and children’s prosocial lie-telling were not associated. However, children’s emotional understanding (which shares considerable conceptual overlap with cognitive empathy), assessed in a story-book task (Pons & Harris, 2000), predicted their prosocial lie-telling. In sum, recent correlational work provides mixed evidence on the link between children’s empathy and their prosocial lie-telling (Demedardi et al., 2021; Nagar et al., 2020).

Most researchers studying children’s empathy and prosocial lie-telling assume that the correlational link between them is causal. Nagar et al. (2020) posit that cognitive empathy is an “underlying cognitive process” (p. 1) and “prerequisite” (p. 14) of prosocial lies. Similarly, Demedardi et al. (2021) state, “to decide to tell a prosocial lie to help a person in distress, a child must be able to recognize the emotion of the person whom the lie benefits […]” (p. 5). However, rigid tests of causal claims remain rare.

Some researchers aimed to induce empathy by manipulating the affective states of children’s interlocutors (Nagar et al., 2020; Warneken & Orlins, 2015; Williams et al., 2014; see also Xu et al., 2019). However, the role of empathy in these manipulations remains speculative unless tested explicitly. Reacting to others’ sad affective states may be driven by politeness norms (Brown, 2015) or guilt (Vaish, 2018; Vaish et al., 2016) rather than empathy.

Short-time interventions offer a promising pathway to test, rather than assume, causality. Experimentally increasing or decreasing empathy and observing pre- and post-intervention modulation in behavioral responses provides a critical test of causal associations between empathy and prosocial behaviors, such as polite lie-telling. To date, as far as we know, short-term interventions fostering children’s empathy are absent. While some kindergarten- and school-based intervention programs target empathy, these programs are typically administered over extended periods ranging from multiple days to several months. Thus, their applicability in developmental science research remains limited (e.g., Brazzelli et al., 2021; see Malti et al., 2016 for a review).

### The Current Study

The current study introduces a novel short-time story book-based empathy intervention to test the causal effect of empathy on children’s prosocial behaviors in politeness dilemmas (Warneken & Orlins, 2015). That way, we aim to shed light on the motivational underpinnings and potential explanations for the observed developmental shift in children’s reactions to politeness situations (Jakubowska et al., 2021; Warneken & Orlins, 2015).

The intervention focuses on affective perspective-taking (Decety & Cowell, 2014) and the empathic inference and adoption of a story character’s feelings based on the four basic emotions of sadness, happiness, anger, and fear (Ekman & Friesen, 1971). The design of the intervention was inspired by evidence-based school programs such as the Second Steps Program (Committee for Children, 1989) or the Incredible Years School Dinosaur (Webster-Stratton, 2011; see Malti et al., 2016 for a review; see also Voltmer & von Salisch, 2022). Moreover, relying on stories and conversations about emotions has proven efficient in fostering toddlers’ empathic skills (Brazzelli et al., 2021). We developed a parallel control intervention to evaluate the intervention’s effectiveness. Besides, we tested children’s empathy at baseline (pre-intervention) and following the intervention (post-intervention) using a conceptually similar picture-story-based task closely aligning with well-established empathy assessments for children in the current age range (Ricard & Kamberk-Kilicci, 1995; see Method section for details).

We used an adapted version of the art-rating task (Fu & Lee, 2007; Warneken & Orlins, 2015) to assess children’s polite lie-telling and prosocial encouragement. Testing children’s empathy in story-book tasks while assessing their prosocial reactions in a distinct live interaction allowed us to dissect the targets of children’s empathy and prosociality to tap the assumed causal links regardless of interindividual relationships (i.e., being prosocial to individuals one empathizes with). To control for potential links between children’s executive functioning and their polite lie-telling behaviors (Talwar et al., 2017; Williams et al., 2016), we included a day-night Stroop task (Gerstadt et al., 1994) in the study procedure.

We tested children from urban Germany. German caregivers emphasize their children’s independence and self-reliance, forming part of an autonomous cultural milieu (Kärtner, 2015). Children’s psychological autonomy and independence become increasingly emphasized throughout the elementary school years (Rimm-Kaufman & Pianta, 2000). Adults encourage children to navigate social interactions based on their subjective preferences rather than strict social obligations (Kärtner, 2015). Attachment parenting presents a widespread ideal in childrearing such that caregivers often aim to respond to their children’s needs empathically. Parents consider empathy an essential developmental task and aim to promote their children’s empathic development (Brisch & Hollerbach, 2018). We discuss how the urban German socio-cultural context may have affected children’s performance in the current study in a dedicated constraints on generality section in the discussion (Simons et al., 2017).

We included children between 5 and 8.5 years, as previous research suggests a developmental increase in prosocial lie-telling in middle childhood (Jakubowska et al., 2021; Warneken & Orlins, 2015). Generally, this age marks a crucial period for studying prosocial behavior as children increasingly shift from self- to other-oriented perspectives (e.g., Blake et al., 2015). Indeed, previous research suggests that children’s prosocial lies become increasingly other-oriented throughout middle childhood (Popliger et al., 2011; Xu et al., 2010).

We preregistered hypotheses 1–3 based on a stepwise data analytic approach (https://osf.io/758c3). However, after testing six participants, we noticed that some responded to the art-rating task by cheering the artist up and providing prosocial encouragement (see Warneken and Orlins (2015) for a similar observation). We thus decided to code this behavior (i.e., prosocial encouragement; pre-registered as “encouraging truth-telling”) as an additional proxy for prosocial behavior and amended our preregistration accordingly by adding hypotheses 4–7 (https://osf.io/52sqx).

First, we predicted that participating in the empathy intervention would increase children’s empathy (*Hypothesis 1*). Accordingly, we expected a more significant increase in empathy scores from pre- to post-intervention for children in the empathy than in the control condition.

If this was true, our second hypothesis assumed a promotive effect of the intervention on children’s polite lie-telling (*Hypothesis 2*), on children’s tendency to be encouraging (*Hypothesis 4*), and their general tendency to react prosocially (i.e., display at least one of these two behaviors; *Hypothesis 6*). Thus, children in the empathy condition should be likelier to tell polite lies, provide prosocial encouragement, and thus generally react more prosocially than children in the control condition following the intervention. Based on Warneken and Orlins (2015), we further assumed that these links might become more pronounced with age throughout middle childhood.

Further, regardless of the intervention’s effect, we assumed that children’s baseline empathy, assessed before the intervention, would predict their polite lie-telling (*Hypothesis 3*), their prosocial encouragement (*Hypothesis 5*), and their combined prosocial behaviors (*Hypothesis 7*). More specifically, we expected children with higher baseline empathy to be likelier to tell polite lies, be encouraging, and react more prosocially than their peers with lower scores. While strict causal inference regarding this link suffers from the non-experimental assessment of these variables, we proposed a causal model using directed acyclic graphs (Pearl, 2013), following the suggestions of Rohrer (2018; see OSF repository: https://osf.io/7pvrx). Again, we assumed that such links should become stronger with age (Warneken & Orlins, 2015).

Since the merged analysis of the combined prosocial outcome (*Hypotheses 6 and 7*) is redundant with the analyses of both prosocial behaviors separately, we moved the details of these analyses and results to the supplementary material (see OSF repository).

## METHOD

### Participants

We tested 115 children but excluded 19 from statistical analyses because they had sorted the distractor drawings incorrectly in the polite lie-telling phase (*n* = 12) or because of experimenter error (*n* = 7). Experimenter errors entailed forgetting to read one of the stories or not asking all test questions such that children could not receive the full score (*n* = 4) or accidentally testing children in a wrong condition according to our counterbalancing scheme (*n* = 3). The final sample comprised *N* = 96 predominantly White children aged between 5.0–8.5 years (*M* = 6.92; *SD* = 1.10; girls = 48, boys = 48). Data collection took place between October to December 2020 and August to December 2021 (with the gap between both periods being due to testing restrictions in response to the COVID-19 pandemic).

We recruited children from a participant database at the Max Planck Institute for Evolutionary Anthropology in Leipzig (Saxony), Germany. All children came from Leipzig or surrounding areas, a German city with about 600,000 inhabitants. We did not collect individual socioeconomic variables, but families in this database typically come from mid-to-high socioeconomic backgrounds. Families in Saxony are usually small and nuclear, with an average of 1.56 children per mother (Statistisches Bundesamt, 2019). Urban German households typically comprise two generations living together (Otto & Keller, 2015). Usually, parents have high levels of formal education and work in professional jobs integrated into a market economy. Children enter formal schooling at around six years of age, following some years in daycare institutions. Schooling takes place in age-homogenous groups under the supervision and tuition of teachers.

### Power Analysis

An a priori power analysis suggested a sample size of 88 to 128 participants to obtain a power of .80. As a compromise between the recommended number of participants, resource constraints, and the pandemic situation in 2020 and 2021, we preregistered a sample size of *N* = 96 children (i.e., 48 children per condition). Specifically, when estimating the required sample size for evaluating the intervention’s effectiveness (i.e., the impact of *condition* on the *change in empathy*), we simulated data and focused on the p-value of the respective full-null model comparisons, assuming a medium effect of *condition*. For the impact of *condition* and *age* on the probability of telling a polite lie, we again focused on the p-value of the respective full-null-model comparisons, assuming a medium effect of *condition* and a *small* effect for age. Lastly, for analyzing the impact of *baseline empathy* on the probability of telling a polite lie, we again focused on the p-value of the respective full-null model comparisons. Here, we assumed small effects of *baseline empathy* and *age* (see script provided in OSF repository).

### Experimental Design

We randomly assigned children to one of two conditions. Hereby, we followed a stratified randomization scheme regarding gender and age to ensure there was no systematic variation between children in the two conditions (see OSF repository for descriptive statistics per condition). In the empathy condition, a female experimenter (E1) modeled empathy with the respective story protagonists. In the control condition, E1 reflected on objects and non-empathic content in the stories. We assessed children’s empathy before and after this manipulation with a novel empathy task. Additionally, a subset of caregivers (*n* = 75) rated their children’s empathy using a German version of the Griffith Empathy Measure (GEM; adapted from the Bryant’s Index of Empathy (Dadds et al., 2008; German version by Greimel et al., 2011; see also Demedardi et al., 2021; Nagar et al., 2020). We further assessed children’s executive functioning (EF) utilizing a day-night Stroop task (Gerstadt et al., 1994) to control for the effects of inhibitory control on polite lie-telling (Talwar et al., 2017; Williams et al., 2016). Lastly, we observed children’s behavior in an art-rating task (Jakubowska et al., 2021; Warneken & Orlins, 2015).

### Materials

Two cameras recorded children’s behaviors and responses in the study room. Per child, we used three DIN A5-story books. The respective illustrations and standardized instructions matched the child’s gender. The first book was used to assess empathy at baseline (i.e., pre-intervention). The second book served as a medium for the experimental manipulation (i.e., intervention). The third book assessed children’s empathy after the experimental manipulation (i.e., post-intervention). Each book contained four stories with two to three illustrations each. Respectively, two of the stories depicted a single emotion (pre- and post-intervention: fear, anger; intervention: happiness, sadness). The remaining two stories depicted more complex situations with sequentially felt emotions (pre- and post-intervention: sadness-happiness; intervention: happiness-sadness) and with simultaneously felt emotions (all books: fear and happiness). The emotion combinations were the same for both empathy assessments (pre- and post-intervention) to ensure a similar level of difficulty. However, we chose different emotion combinations in the intervention phase so that children did not improve their performance by merely imitating or copying E1’s behavior. Each story started with an emotionally neutral picture, and we separated stories with blank slides. The task structure followed the protocol of Ricard and Kamberk-Kilicci (1995; for details, see OSF repository). We counterbalanced the order of the stories by *condition* and *gender*.

**Figure 1.**
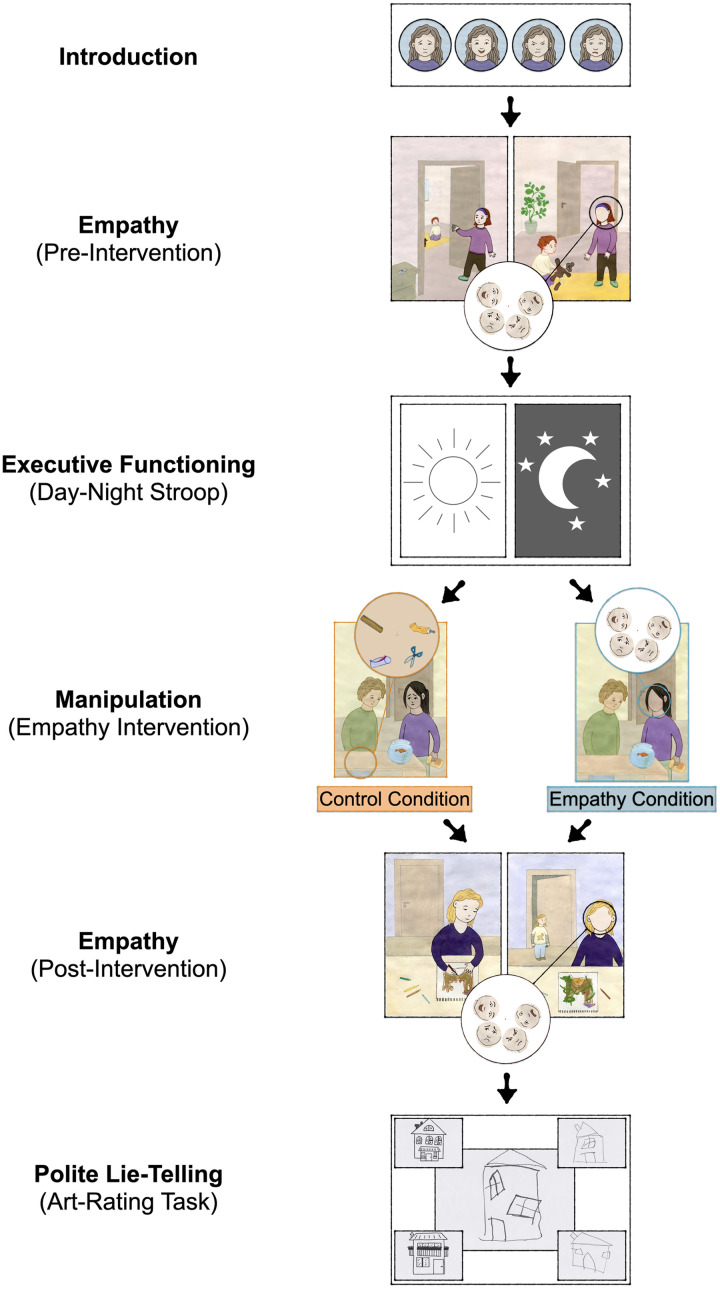
Study design. *Note*. Exemplary story vignettes are depicted to illustrate empathy assessment at pre- and post-intervention and the empathy intervention.

In a novel response format, children could turn a wheel attached to the respective page to set the character’s presumed emotional facial expressions (see Figure 1). Each wheel’s default setting lacked any facial expressions. Instead of simple emotion labeling, children thus had to focus on the situation and infer the protagonist’s inner experience (Ricard & Kamberk-Kilicci, 1995). We gave children a sheet depicting all four emotional expressions (henceforth: emotion overview) following the Facial Action Coding System (FACS; Ekman & Friesen, 1975; see Figure 1). For the day-night Stroop task, we used 14 cards depicting either a sun (7 cards) or a moon with stars (7 cards; Gerstadt et al., 1994).

In the art-rating task, we used two transparent stacking trays (26 × 35 × 7 cm) on which children could sort the drawings. We counterbalanced the sides of the respective good and bad piles (left; right). For the training phase, we used four photos of vases, two of which were broken and two of which were intact. In the testing phase, we used five drawings of houses (two of good quality and three of bad quality, including the target drawing of bad quality). To resolve potential tension and leave children feeling good, the artist presented an additional drawing of good quality (i.e., a car) after the testing phase.

### Procedure

Children were invited to the laboratory. We tested all children and their caregivers for COVID-19. The experimenters wore face masks throughout the study. E1 conducted a brief warm-up phase with the child by discussing hobbies, school, or holiday plans. Next, she introduced a second female experimenter (E2) to the child. She mentioned that E2 would join them later but draw some pictures in the meantime. We kept E2 blind to the child’s condition throughout the study. Next, E1 invited the child to a separate study room, where both sat at a table (see Figure 1 for an overview of the procedure; for details of the preregistered procedure, see OSF repository).

#### Caregiver Involvement.

In the child’s absence, E2 asked the caregivers to fill in an empathy questionnaire and briefed them about the art-rating task their child would face. We instructed caregivers to ask their children about E2’s drawing as an additional proxy for their ratings upon return. Caregivers received a template with the printed instruction “Please ask your child about the study and whether they liked E2’s drawing of the house” and two check-boxes (“yes”; “no”).

#### Introduction.

In the study room, E1 introduced the four emotions depicted in the emotion overview (i.e., sadness, happiness, anger, and fear). E1 pointed out the salient features of the corresponding facial expressions by focusing on the eyes and mouth regions. Next, E1 introduced a drawing of a gender-matched child and an attached emotion wheel and asked the child to set each of the four emotions.

#### Empathy (Pre-Intervention).

E1 read the first story and asked the child to retell the story briefly (i.e., comprehension check). If the child failed to mention predefined keywords referring to the story, E1 read the story a second time and asked the child to retell the story. In case the second attempt failed, E1 continued with the following story. E1 reminded the child that there were no right and wrong answers and that they could reply freely to reduce social desirability demands.

E1 asked three questions per story, addressing children’s emotion ascription (“How does [protagonist’s name] feel?”), their emotional reactivity (“How does [protagonist’s name] make you feel?”), and their perspective-taking (“Why does [protagonist’s name] make you feel that way?”; see Ricard & Kamberk-Kilicci, 1995). The child could set the protagonists’ emotional expressions via the emotion wheel attached to respond to the first question. They could address the second question verbally or point to a respective image on the emotion overview. The third response had to be given verbally. E1 followed this protocol for all four stories.

#### Executive Functioning (Day-Night Stroop).

E1 proceeded with a day-night Stroop task based on Gerstadt et al. (1994) to assess the child’s inhibitory control. E1 showed the child a (black) moon card and told them to say “day” when seeing such cards. Next, she flipped the (white) sun card and asked the child to say “night”. E1 tested the child’s comprehension by first showing the white sun card and then the black moon card. Pending the child’s comprehension, E1 presented the remaining cards in a pseudorandomized sequence (night (n), day (d), d, n, d, n, n, d, d, n, d, n, n, d).

#### Manipulation (Empathy Intervention).

**Figure 2.**
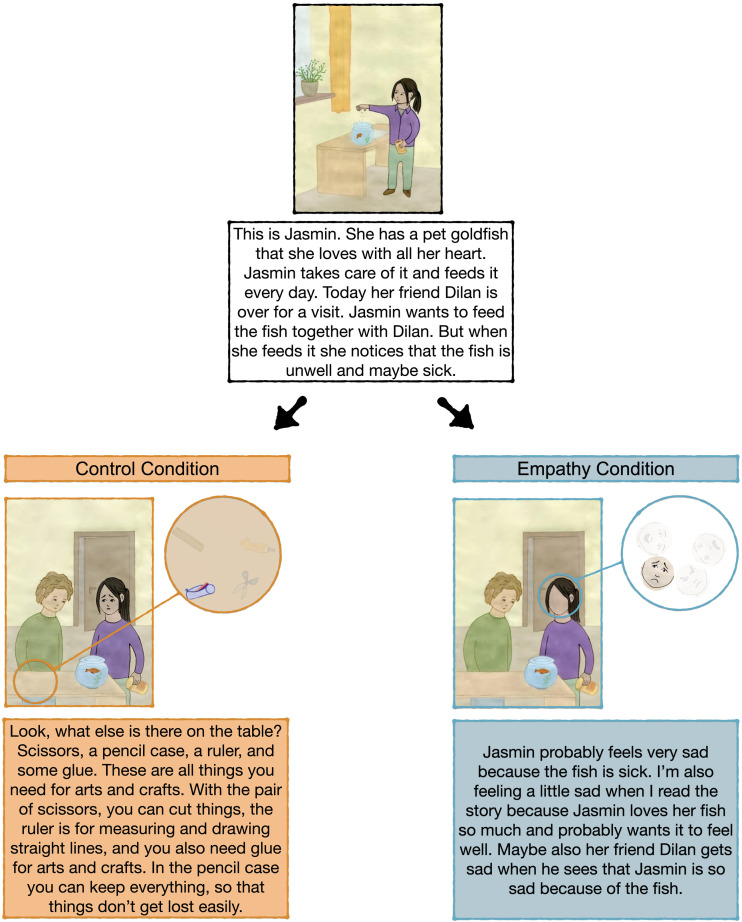
Exemplary story vignette in empathy intervention (control condition, empathy condition). *Note*. Exemplary story vignettes illustrating the empathy and control condition. The illustration depicts the response to a single emotion (sadness) in the empathy condition and a response (pencil case) in the control condition.

E1 stated that now it was her turn to set the wheels. Like the empathy task at pre-intervention, she read each story aloud and ensured the child’s comprehension of the story’s content. E1 presented the same stories and pictures across conditions. However, in the empathy condition, the wheels depicted the protagonists’ emotional expressions (see Figure 2). Throughout this intervention phase, E1 responded highly empathetically to the four stories. Parallel to the empathy task, E1 first inferred how the specific context emotionally affected the respective protagonist and used the attached wheel to set their facial expression. Next, she modeled empathy by stating how she felt (i.e., displaying a similar emotion as the protagonist). Lastly, she related her feelings to the protagonist’s emotional or inner state (for details, see OSF repository). We did not inform children that this served as a modeling phase.

The control condition deviated from this protocol in that E1 did not refer to the emotions of the story protagonists. Here, the protagonists had emotionally neutral facial expressions throughout the stories. Further, the wheels were attached to other regions in the illustrations such that E1 could place objects into the scenes by turning the wheels (e.g., food in the fridge, toys, craft items). E1 inspected and described all items before choosing one of them.

Following the experimental manipulation, E1 briefly left the study room to check on E2. This served as a short break for the child and a reminder that E2 was still composing a drawing in the adjacent room.

#### Empathy (Post-Intervention).

For better comparability between study phases, we confronted the child with identical emotion combinations (i.e., fear, anger, sadness-happiness, fear-happiness), instructions, and order of the stories as in the pre-intervention phase. However, we introduced new storylines and protagonists to avoid carry-over effects.

#### Polite Lie-Telling (Art-Rating Task).

The procedure of the polite lie-telling task was based on Warneken and Orlins (2015). For familiarization with the task procedure, E1 asked the child to help her sort photos of vases according to their apparent quality into a good and a bad pile. She indicated which tray would be for the good ones and which for the bad ones. As the child began sorting, E1 stated she had to check something in the hallway but would return quickly (i.e., when the child had sorted all vases). When the child was done sorting, E1 checked whether their sorting was correct. If not, each child was given a maximum of three attempts to sort different sets of vases to ensure comprehension of the sorting task.

Next, E1 proceeded with the testing phase. According to the established rule, she asked the child to sort four drawings of houses (two of good and two of bad quality). Again, she left the room as the child started but said she would return quickly. When the child was done, E1 returned with E2, who stayed at the door holding her bad drawing of a house (the drawing facing herself, not the child). E1 asked E2 how she felt, and E2 replied she was very sad, as she had tried to compose a good drawing but failed to do so. In line with the original procedure, E1 whispered that E2 was indeed very sad and that maybe the child could think of something to make her feel better (Warneken & Orlins, 2015). Again, E1 excused herself and left the room. E2 sat down at the table opposite the child. She noticed the piles and showed her drawing. Next, she asked the child on which pile they would sort her drawing (i.e., target drawing). If the child pointed to one of the piles, E2 asked whether this was the good or the bad pile. Next, she placed her picture in the center between the piles and stated she might continue with that drawing later. E2 left the room while E1 returned. She asked the child how they liked E2’s drawing before leaving the study room with the child. Outside they met E2, who proudly announced that she had composed another drawing (i.e., car) that turned out very well.

In the absence of E1 and E2, caregivers asked the child about the study and how they liked E2’s previous drawing of a house. Finally, E1 thanked the child for participating, and the child could pick a gift to recompensate them for their participation.

### Coding

Student assistants, otherwise uninvolved in the research and unaware of hypotheses, coded all data from tape or transcribed utterances. The first author coded an additional 33 videos (i.e., 34%) to estimate interrater reliability.

#### Empathy (Pre- and Post-Intervention).

We transcribed all verbal responses and coded them based on the scheme of Ricard and Kamberk-Kilicci (1995) into a match- and an interpretation score. The match score estimated to which extent children’s emotions matched those of the story’s protagonist (i.e., 0 = no match, 1 = same valence, 2 = precise match). The degree of the match was determined based on children’s responses regarding the emotion ascribed to the story protagonist (emotion ascription) and the emotion that they reported feeling themselves (emotional reactivity). The interpretation score served as a proxy for children’s perspective-taking by evaluating the reasons children provided for their emotional experience (i.e., 0 = no relation to the story; 1 = descriptive reasoning; 2 = general or child-centered reference to an emotion or inner state; 3 = character-centered reference to an emotion or inner state). Each child could score between 0 and 5 points per story, resulting in 20 points per story-book (for details, see OSF repository). By acknowledging different aspects of the children’s responses in the coding, we follow previously validated methods (Ricard & Kamberk-Kilicci, 1995) and receive a final score that reflects both the affective (match) and cognitive (interpretation) dimensions of empathy. Interrater reliability was excellent regarding the pre-intervention (*ICC* = .98) and the post-intervention empathy task (*ICC* = .97).

#### Executive Functioning (Day-Night Stroop).

We coded children’s executive functioning by the number of cards they labeled correctly in the day-night Stroop task. Children received a score between 0 and 14. Interrater reliability was perfect (*ICC* = 1).

#### Polite Lie-Telling and Prosocial Encouragement (Art-Rating Task).

We coded children’s sorting of the target drawing onto the bad pile as truth (0) and the good pile as a lie (1). Besides, we transcribed children’s complete responses and coded whether these contained any encouragement or cheering up (1) or whether such utterances were absent (0) - regardless of whether these children had previously told the truth or a lie. We further noted whether children had rated the drawing as good (1) or bad (0) in the presence of E1 and their caregivers, respectively. Interrater agreement for both variables was excellent (polite lie-telling: *κ* = 1; prosocial encouragement: *κ* = .90).

### Statistical Analyses

We preregistered all scripts and analyses on the Open Science Framework (see OSF repository). We scaled all continuous predictors to a mean of 0 and a standard estimation of 1. To avoid inflation of type 1 error, we ran full-null model comparisons (Forstmeier & Schielzeth, 2011). Assumption checks and model stability parameters indicated no problematic issues (see OSF repository).

For the models investigating the intervention’s effectiveness and the causal impact of empathy, we preregistered to include a 2-way-interaction between *condition* and *age* into the full model if an initial analysis would reveal a statistically significant effect. However, the respective full-null model comparisons or the specific estimates of the interaction terms did not indicate such effects. Thus, we continued investigating full models comprising main effects only.

#### Intervention’s effectiveness (Hypothesis 1).

First, we assessed if children in the empathy condition showed a greater increase in empathy from pre- to post-intervention than those in the control condition. We modeled the effect of our test predictor *condition* [Control; Empathy] on *change in empathy* [−20–|20|] by fitting a linear model with a Gaussian response distribution. Besides, we included children’s *age* [5–8.5], *gender* [Female; Male], *EF* [0–16], and *pre-intervention empathy* [0–20] as controls.

#### Causal Impact of Empathy (Hypotheses 2 and 4).

Since *condition* had a statistically significant impact on the change in children’s empathy (see Results below), we assessed whether children in the empathy condition were more likely to engage in *polite lie-telling* [0; 1] (*Hypothesis 2*) and *prosocial encouragement* [0; 1] (*Hypothesis 4*) than those tested in the control condition. In addition to *condition*, we included *age* [5–8.5] as a test predictor in both models, as previous research indicated an increase of prosocial lies with age (Warneken & Orlins, 2015; Xu et al., 2010). Respectively, we fitted a generalized linear model with a binomial response distribution and logit link function to estimate the impact of *condition* and *age* on the probability of engaging in these behaviors. Furthermore, we controlled for children’s *gender* [Female; Male], *EF* [0–16], and *pre-intervention empathy* [0–20].

#### Impact of Pre-Intervention Empathy (Hypotheses 3 and 5).

In addition to analyzing the impact of fostering children’s empathy on their behavior in the art-rating task, we inspected the effect of children’s *pre-intervention empathy* on both prosocial behaviors. We decided to add this explorative analysis since the first analysis indicated that children with higher empathy scores at pre-intervention benefited only marginally from the intervention. Causal interpretations of this explorative analysis should only be made with caution since children’s *pre-intervention empathy* was not assessed experimentally. For clarity, we provide causal acyclic graphs to document the expected causal effect of empathy on prosociality in politeness contexts (see OSF repository).

Here, we assessed whether children’s *pre-intervention empathy* [0–20] was associated with children’s *polite lie-telling* (*Hypothesis 3*) and *prosocial encouragement* (*Hypothesis 5*). We included *age* [5–8.5] as a further test predictor. We fitted two generalized linear models with a binomial response distribution and logit link function to estimate the impact of *pre-intervention empathy* and *age* on the respective outcome. Further, we controlled for children’s *gender* [Female; Male] and *EF* [0–16].

#### Reliability and Validity Empathy Task.

To assess the reliability of the novel empathy task, we calculated the correlations between children’s *pre-intervention empathy* and *post-intervention empathy* in each condition.

Moreover, we inspected the correlations between children’s *age* and performance in the *pre- and post-intervention empathy* assessment, respectively.

To evaluate the task’s validity, we calculated correlations between children’s *pre-intervention empathy* and caregiver-rated empathy relying on the GEM (Dadds et al., 2008; German version by Greimel et al., 2011). Respectively, we investigated the correlations between children’s *pre-intervention empathy* and the *total GEM* score, the *affective empathy subscale* score, and the *cognitive empathy subscale* score. These correlations included a subset of the data (n = 75), as the GEM was only administered to a fraction of children’s caregivers.

### Transparency and Openness

We report how we determined our sample size, give reasons for the cases we dropped from the analyses, provide a detailed description of all manipulations and measures in the study, and follow the Journal Article Reporting Standards (Kazak, 2018). Our preregistration, data, code for the analysis, and detailed supplementary materials supporting this study’s findings are available on the Open Science Framework (https://osf.io/7pvrx). Included are copies of all study materials allowing interested scholars to apply the empathy task and intervention in their research. We conducted all analyses in R, version 4.2.1 (R Core Team, 2022).

## RESULTS

### Intervention’s Effectiveness (Hypothesis 1)

**Figure 3.**
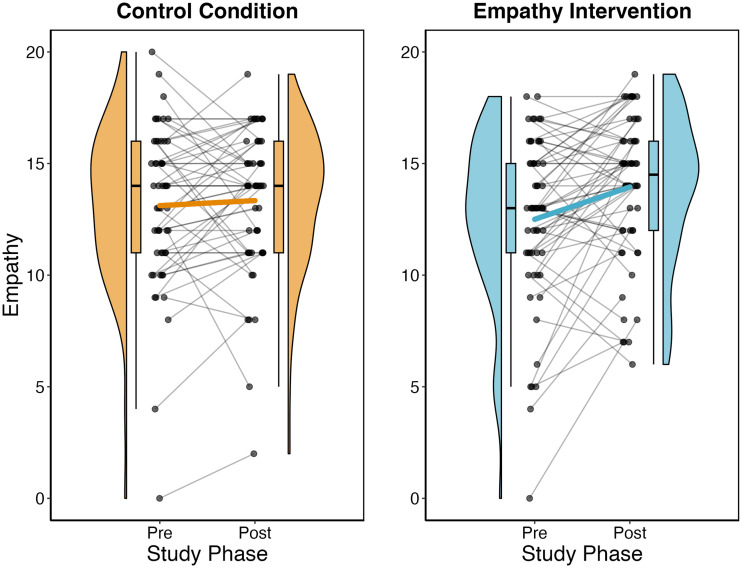
Effects of the empathy intervention on children’s empathy from pre- to post-intervention. *Note*. Children’s empathy scores across conditions and study phases. Black dots represent data points for each study phase; black lines link individuals across study phases. Blue (right panel) and yellow lines (left panel) link means across study phases; violin plots indicate kernel probability densities of the data. Box plots indicate medians (black horizontal lines) and interquartile ranges (boxes) for each study phase and condition; data is jittered on the x-axis for better visualization.

*Condition* had a statistically significant effect on children’s change in empathy from pre- to post-intervention (full-null model comparison: *F*_1,90_ = 6.28, *p* = .014). Specifically, participating in the empathy intervention (*M*_*Change Empathy Intervention*_ = 1.48, *SD* = 3.39) but not the control condition (*M*_*Change Control Condition*_ = −0.08, *SD* = 2.82) resulted in an increase in empathy (*estimate* ± *SE* = 1.35 ± 0.54, *t* = 2.51, *p* = .014, *partial R*^2^ = .07; see Figure 3).

Inspection of the model parameters also revealed that children who displayed higher empathy at pre-intervention showed smaller increases in empathy from pre- to post-intervention as compared to children with lower empathy at baseline (*estimate* ± *SE* = −1.96 ± 0.31, *t* = −6.40, *p* < .001, *partial R*^2^ = .31). Neither of the remaining control predictors (i.e., *age*, *gender*, *EF*) had a statistically significant effect on children’s changes in empathy from pre- to post-intervention (for details, see OSF repository).

### Causal Impact of Empathy (Hypotheses 2 and 4)

**Figure 4.**
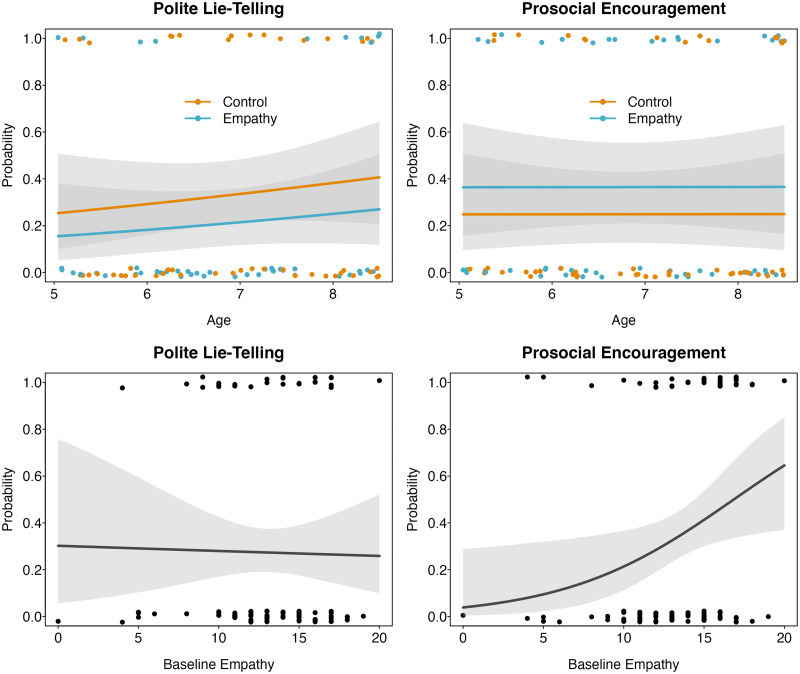
Relation between children’s empathy and their prosocial behaviors. *Note*. Fitted values of model estimates for the effects of condition and age (Hypotheses 2 and 4, upper panel) and the effects of children’s baseline empathy at pre-intervention (Hypotheses 3 and 5). Grey areas depict 95%-CIs; data are jittered on the y-axis for better visualization.

A full-null model comparison did not indicate a statistically significant effect of our test predictors *condition* and *age* on children’s polite lie-telling (*χ*^2^ = 2.34, *df* = 2, *p* = .310, *Nagelkerke’s R*^2^ = .04) or their prosocial encouragement (*χ*^2^ = 1.48, *df* = 2, *p* = .478, *Nagelkerke’s R*^2^ = .02). We found no evidence that children would tell more polite lies following participation in the empathy intervention (20.83%) compared to the control condition (33.33%). Similarly, we found no indication that children were more encouraging following the empathy intervention (39.58%) than the control intervention (29.17%). Thus, we found no support for a short-time causal effect of empathy on prosocial behavior, as indicated by children’s polite-lie telling or prosocial encouragement (see Figure 4). For details regarding the descriptive statistics, see OSF repository.

### Impact of Pre-Intervention Empathy (Hypotheses 3 and 5)

Given the negative association between children’s pre-intervention empathy and their *change in empathy* from pre- to post-intervention (see above), we decided to explore the link between children’s *pre-intervention empathy* and their prosocial behaviors. Since we did not manipulate children’s *pre-intervention empathy* experimentally, these analyses, as most prior research, do not allow for strict causal inference. However, as there are inconsistent findings regarding the assumed link in recent research (Demedardi et al., 2021; Nagar et al., 2020), we believe that further data linking empathy and polite lies or prosocial encouragement may prove helpful.

A full-null model comparison did not indicate a statistically significant effect of our test predictors *pre-intervention empathy* and *age* on the probability of telling a polite lie (*χ*^2^ = 0.70, *df* = 2, *p* = .706, *Nagelkerke’s R*^2^ = .01). However, *pre-intervention empathy* and *age* had a statistically significant effect on children’s *prosocial encouragement* (*χ*^2^ = 7.34, *df* = 2, *p* = .026, *Nagelkerke’s R*^2^ = .10). More specifically, children with higher pre-intervention empathy scores were more likely to be encouraging in the art-rating task (*estimate* ± *SE* = 0.69 ± 0.30, *z* = 2.30, *p* = .013, *Nagelkerke’s R*^2^ = .09). *Age* (*estimate* ± *SE* = 0.01 ± 0.25, *z* = 0.04, *p* = .965, *Nagelkerke’s R*^2^ = <.001) and neither of the control predictors (i.e., *gender*, *EF*) had a statistically significant effect on children’s *prosocial encouragement.* For details on the analyses, see OSF repository.

Taken together, we did not find support for a link between *pre-intervention empathy* and polite lie-telling (*Hypothesis 3*). However, in line with *Hypothesis 5*, our results indicate a positive association between children’s pre-intervention empathy and their tendency to be encouraging.

### Reliability and Validity Empathy Task

In both conditions, children’s *pre-intervention empathy* was moderately to strongly associated with their *post-intervention empathy* (empathy condition: *r* = .55, *p* < .001; control condition: *r* = .65, *p* < .001). Besides, children’s *age* was weakly to moderately associated with their performance in the empathy tasks (*r*_*age*, *pre-intervention*_ = .41, *p* < .001; *r*_*age*, *post-intervention*_ = .38, *p* < .001).

When linking children’s empathy at pre-intervention to their caregiver-rated empathy, the correlation between *pre-intervention empathy* and the *total GEM score* revealed no statistically significant link between both variables (*r* = .18, *p* = .131). Also, the correlations between *pre-intervention empathy* and the *affective empathy subscale* (*r* = .03, *p* = .828) and *pre-intervention empathy* and *cognitive empathy subscale* (*r* = .16, *p* = .169) indicated no statistically significant association, respectively.

## DISCUSSION

Children’s empathy is commonly considered a driver of prosocial behavior in early and middle childhood. Still, such claims are limited, given the prevalence of research designs that allow for correlational but not causal inference. Here, we introduce a short-term empathy intervention enabling a more rigorous study of empathy’s short-time causal effects on developmental outcomes. In line with previous research (Eisenberg & Miller, 1987; Pavey et al., 2012), we found an association between children’s empathy at baseline and their encouraging behavior as a proxy for prosocial behavior. Furthermore, children who participated in the empathy intervention but not the control condition indicated a significant increase in their empathy from pre- to post-intervention empathy assessment. However, this promotive effect of the empathy intervention did not lead to increased prosociality in an art-rating task. Children in the empathy condition were not more likely than children in the control condition to engage in polite lie-telling or prosocial encouragement.

### An Intervention to Study Causal Effects of Empathy in Developmental Psychology

The current study introduces and evaluates a novel short-time intervention to foster children’s empathy within experimental settings. This intervention approach enables a more direct examination of empathy’s effects on prosocial behaviors compared to previous work that assessed both phenomena in non-experimental research designs (Demedardi et al., 2021; Nagar et al., 2020) or that manipulated partners’ affective states as a proxy for children’s empathy (Warneken & Orlins, 2015).

Against our expectations, we did not find a statistically significant link between children’s empathy at baseline as assessed with the novel task and the caregiver reports of children’s empathy (Greimel et al., 2011). However, the stories we implemented in the empathy intervention and assessment closely align with those used and validated in previous research (Ricard & Kamberk-Kilicci, 1995). Further, it is worth noting that such dissociations between sources of information have been reported for other well-established measures of developmental outcomes (Huber et al., 2019). Given the practical and epistemological benefits of assessing developmental outcomes by directly asking children rather than caregivers (Huber et al., 2019), we believe the current assessment and intervention offer a promising approach for future investigations of empathy and its causal effects on social development.

Notably, children’s performance in the empathy tasks indicated considerable reliability between pre- and post-intervention assessment in both conditions. This illustrates systematic interindividual variation in children’s empathetic responding that can be reliably captured with the current measure. As such, scholars may simultaneously study its causal and correlational associations with prosocial behavior and other developmental outcomes. Further, the association between children’s age and performance in the empathy assessment indicates that the task captures children’s developing empathic skills with age.

We found that children’s empathy at baseline was associated with their tendency to be encouraging. Causal interpretations of this link are limited, as children’s baseline empathy was not experimentally manipulated. To complement previous research, we propose a causal model based on previous research and theory as a provisional framework (see OSF repository). Acknowledging that more research is needed to further validate the current approach, we highlight the benefits of the novel intervention for the developmental study of empathy within causal frameworks. We provide relevant materials, including illustrations and scripts, alongside this manuscript to support the application and critical evaluation of the current empathy intervention and assessment (see OSF repository).

### Linking Empathy to Polite Lie-Telling and Prosocial Encouragement

Our findings are partly in line with prior correlational evidence for links between empathy and prosociality (Batson, 1997; Eisenberg & Miller, 1987): Children’s empathy at baseline predicted their prosocial encouragement but not their polite lie-telling. One might speculate whether polite lie-telling and prosocial encouragement differ in their motivational underpinnings. Prosocial encouragement (similar to comforting) might present a more other-directed and genuinely prosocial strategy to navigate politeness dilemmas in early childhood. Such behaviors may become increasingly strategic in mid- to late childhood as children use them to manage their reputations (Grueneisen & Warneken, 2022). Polite lies, in contrast, may comprise a more dynamic mixture of motives early on in childhood (Popliger et al., 2011; Talwar & Lee, 2002; Xu et al., 2010), including prosocial motives but also motives such as conflict avoidance, norm adherence, and reputation management. Besides, one might speculate whether the relation between empathy and polite lie-telling is more complex as social and cultural learning might affect peoples’ readiness to engage in such behaviors (Giles et al., 2019). Future research is needed to disentangle these motives as drivers of polite lie-telling.

Besides, participating in the empathy intervention did not increase prosocial behaviors in the subsequent art-rating task. Children following the empathy intervention did not utter more polite lies or more prosocial encouragements than children in the control condition. Thus, the current study adds to and extends previous research, raising doubt on an immediate causal link between empathy as a driver of children’s prosocial lie-telling (Demedardi et al., 2021) and prosocial behavior more generally. We do not find support for empathy as a short-term, immediate switch to foster children’s prosocial reactions. Notably, the absence of a causal link between empathy and polite lie-telling and prosocial encouragement in the current study might not generalize to prosocial behaviors in other contexts, given the diversity of prosocial behaviors, their motives, and the developmental mechanisms involved in this regard (Paulus, 2014).

Further, a causal effect of empathy on polite lies might need more time to fully consolidate than given in the current study - such as by giving children time to navigate the *Maxim of Quality* and the *Politeness Principle* when engaging with their caregivers or friends before applying such tendencies when engaging with strangers. Longitudinal intervention studies may help address such questions in the future.

Moreover, one other possibility is, that the intervention’s effect was not strong enough to affect children’s prosocial behaviors immediately. If so, developing stronger interventions than the current one may provide a causally rigid test of empathy’s effects on children’s prosocial behaviors. To do so, we suggest including younger children in future research, as we observed that children with lower empathy scores at baseline benefitted from the intervention to a markedly greater extent. As such, preschool children younger than those tested here may benefit more from the empathy intervention and may thus be more likely to also increase their prosocial tendencies consequentially.

This is the first study to directly assess the links between children’s empathy and prosocial lies in a politeness context. Here, children needed to ponder the conflicting demands of honesty and harm avoidance. Related work studied children’s lie-telling following an adult’s explicit request (Demedardi et al., 2021; Nagar et al., 2020). To obey authority, children had to lie to a third party about the result of the game outcome. In the current paradigm, prosocial lie-telling may demand more intrinsic motivation to uplift others’ emotions (Warneken & Orlins, 2015).

At first glance, one may argue whether the absence of an association between empathy and polite lies opposes the finding by Warneken and Orlins (2015), who found children being sensitive to their partners’ emotional states when deciding whether or not to tell polite lies. However, it is important to consider that our study did not manipulate the partner’s emotional state but kept it constant following the sadness condition in Warneken and Orlins (2015). Doing so, we sought to rule out behavioral norms (e.g., being nice and comforting to sad people) and situational factors but tap effects of children’s personal-level empathy instead. A second apparent difference between the current paradigm and results and prior work concerns the low rates of polite lies in our study. Warneken and Orlins (2015) found that up to 71% of the 5–8-year-olds engage in polite-lie telling. Here, we found markedly lower rates, with only 27% of children engaging in such behaviors. Despite procedural differences across studies, we believe that cultural variation in politeness norms across the U.S. and Germany may contribute to this discrepancy (Giles et al., 2019; for more details, see Constraints on Generality).

It is further important to note that the current focus on empathy is contrasted by a more diverse range of related phenomena studied in other work: This includes foci on *emotional understanding* (Demedardi et al., 2021), *compassion* (Lupoli et al., 2017) or *empathic concern* (Xu et al., 2019). Investigating causal effects of empathy and its conceptual relatives presents a promising step for future research to provide a more nuanced and concise picture of empathy’s impact on developmental outcomes (Brazil et al., 2022; Jordan et al., 2016).

One limitation of our study is that we cannot fully rule out whether some children coded as polite lie-tellers may have actually liked E2’s drawing. However, we composed E2’s drawing to closely resemble the quality of the two bad distractor drawings. All participants entering this test phase had shown a clear preference to sort these drawings as *bad*, indicating that polite lies not only reflect a more positive tendency in children’s art ratings.

### Constraints on Generality

It is important to note that the current paradigm was designed for and tested in a particular socio-cultural setting. As such, constraints apply when generalizing these findings to children growing up outside urban middle-class milieus in Germany and other countries of the Global North.

We expect our results regarding the empathy task and intervention to generalize to children who have experience with story books, possess such formalized knowledge about emotions as they are felt and verbalized in the current study, and have some experience speaking about their own and others’ emotions. Considering that advanced verbal skills are required to answer the question assessing the reasons why they responded with a particular emotion to a story, the current empathy task and intervention may not be suited well to study children’s empathy in contexts in which such verbalization of emotions is less common as in urban, middle-class milieus in Germany and the Global North.

The observed differences in the prevalence of polite lie-telling between the current study and previous work (e.g., Warneken & Orlins, 2015) stress the importance of considering cultural peculiarities of the study population before generalizing. Specifically, we expect the pattern of results regarding polite lie-telling and prosocial encouragement to vary between contexts with different politeness norms and preferences for directness vs. indirectness (Giles et al., 2019). German communication is typically rather direct and functional, and honesty is encouraged compared to other Westernized societies (e.g., House, 2005; IES, 2021). Even though German adults rate lie-telling in politeness contexts as more acceptable than blunt truth-telling, they find telling the truth in a polite manner more appropriate than telling polite lies. Moreover, German adults prefer honesty over lie-telling (Giles et al., 2019). As such, the base rates of children’s polite lie-telling in the current study may well differ when applying the research design to other cultural communities.

Lastly, it is important to note that polite behavior is a verbal and specific form of prosocial behavior due to the conflicting norms of honesty and politeness. Thus, it remains unclear whether participating in the empathy intervention might affect other forms of prosocial behavior. We have no reason to believe that the results depend on other characteristics of the participants, materials, or context (see Simons et al., 2017).

### Conclusion

Taken together, we do not find support for an immediate, short-time causal effect of children’s empathy on their prosocial behaviors in a politeness context. In the absence of such evidence, our results do not add strong support for a pervasive role of empathy in fostering prosocial lie-telling. This raises some caution on causal claims until those are validated experimentally. We hope the current study will support progress in this scientific agenda by introducing and providing a novel empathy intervention that effectively increases children’s empathy.

## ACKNOWLEDGMENT

We would like to thank Maleen Thiele for helpful comments on an earlier version of the manuscript, Robert Hepach for feedback on the study procedure, Felix Warneken, Emily Orlins, Marcelle Cossette-Ricard, and Ellen B. Braaten for sharing study materials. We thank Jana Jurkat, Leah Kieczkowski, Alexandra Kartashova, Rebekka Franz, Luise Hornoff, Felix Büch, Lukas Willing, Louisa Schmidt, Victoria Gassner, Stefan Gregor, and Hannah Hörl for their support in running the study. Finally, we thank all participating children and caregivers.

## DATA AVAILABILITY STATEMENT

The preregistration, data, code for the analysis, and detailed supplementary materials supporting this study’s findings are available on the Open Science Framework (https://osf.io/7pvrx).

## AUTHOR CONTRIBUTIONS

Noemi Thiede: Conceptualization; Data curation; Formal analysis; Investigation; Methodology; Project administration; Visualization; Writing—Original draft. Roman Stengelin: Conceptualization; Formal analysis; Methodology; Project administration; Visualization; Writing—Original draft. Astrid Seibold: Methodology; Resources; Writing—Review & editing. Daniel B. M. Haun: Funding acquisition; Supervision; Writing—Review & editing.
